# Propranolol and Ivabradine Are Equally Effective in POTS But Not Equipoise in Real Life

**DOI:** 10.1016/j.jacadv.2026.103151

**Published:** 2026-08-13

**Authors:** Ainsley Goff, Catherine Nguyen, Imre Hunyor, John O’Sullivan, Ian Wilcox

**Affiliations:** a Westmead Hospital, Westmead, Australia; b Central Sydney Cardiology, The University of Sydney, Newtown, Australia

The study by Uppal et al^1^ greatly advances the treatment of women with postural orthostatic tachycardia syndrome (POTS), demonstrating a clinically significant reduction in orthostatic heart rate change using propranolol (40-80 mg BD) and ivabradine (5 mg BD) compared to placebo with modest changes in blood pressure. Beyond objective treatment goals, the authors enabled women the preference for either drug at the end of this relatively short-term study.

POTS is a chronic condition, typically recognized years after symptoms begin and is often slow to respond to lifestyle change, physical aids, and pharmacotherapy. Individualized treatment choices should consider concomitant pathologies, symptoms, and life plans. Migraine, including vestibular migraine, headache, and chronic fatigue are prevalent and troublesome. Propranolol has an established role in migraine prevention whereas ivabradine may cause headaches^2^ The beneficial anxiolytic effect of propranolol may be considered important by some women. Although there was no quantitative difference in anxiety assessment between propranolol and ivabradine during the study this may be a time-dependent effect obscured by the short treatment period. As Uppal et al^1^ noted, we find ivabradine may be preferable when baseline blood pressure is low.

An important life choice for these chronically ill women is pregnancy. Pharmacotherapy is not equipoise in pregnancy: propranolol is safe in pregnancy and relatively safe when breastfeeding, whereas ivabradine is not safe in pregnancy but can be used when breastfeeding.^3^

POTS should not be a barrier to family planning. This study positively contributes to the knowledge base of treating POTS, but what it highlights most importantly is the need for patients to exercise choice in their treatment. Restoring some autonomy to women who typically wait years for a diagnosis may act as a therapy of its own.
